# Prenatal Buprenorphine/Naloxone or Methadone Use on Neonatal Outcomes in Michigan

**DOI:** 10.7759/cureus.27790

**Published:** 2022-08-08

**Authors:** Gregory Goshgarian, Rasha Jawad, Laura O'Brien, Robert Muterspaugh, Dimitrios Zikos, Sudhakar Ezhuthachan, Christine Newman, Chaur-Dong Hsu, Beth Bailey, Neli Ragina

**Affiliations:** 1 College of Medicine, Central Michigan University College of Medicine, Saginaw, USA; 2 College of Health Professions, Central Michigan University, Mount Pleasant, USA; 3 Division of Neonatal-Perinatal Medicine, Henry Ford Health, Detroit, USA; 4 Department of Obstetrics and Gynecology, University of Arizona, Tucson, USA; 5 College of Medicine, Central Michigan University College of Medicine, Mount Pleasant, USA

**Keywords:** methadone, buprenorphine and naloxone, neonatal health outcomes, opioid use disorder (oud), neonatal opioid withdrawal syndrome, medication for opioid use disorder (moud)

## Abstract

Background

Maternal opioid exposure during pregnancy has various effects on neonatal health. Buprenorphine/naloxone and methadone are examples of medications for opioid use disorder (MOUD) used for the treatment of opioid use disorder (OUD). Research comparing the impacts of these MOUD modalities on neonatal outcomes when used to treat pregnant people with OUD remains limited. We evaluated the differences in outcomes between neonates with in-utero exposure to buprenorphine/naloxone versus methadone.

Methodology

We performed a retrospective cohort chart review between October 15, 2008, and October 15, 2019, evaluating mother/neonate dyads at two medical centers in Michigan. The charts of female patients, aged 18+, with OUD and buprenorphine/naloxone or methadone treatment, were examined. The charts of the corresponding neonates were also examined. Multiple regression analysis was performed.

Results

In total, 343 mother/infant dyads were included: 99 patients were treated with buprenorphine/naloxone and 232 patients were treated with methadone. The buprenorphine/naloxone group had significant differences in maternal age, hepatitis status, asthma, gestational age in weeks, neonatal intensive care unit (NICU) length of stay (LOS), neonatal opioid withdrawal syndrome (NOWS) peak score, birth head circumference, and birth weight compared to the methadone group at baseline. Adjusted multivariable regression analysis demonstrated neonates with exposure to buprenorphine/naloxone had a NOWS peak score 3.079 points less (95% confidence interval (CI): -4.525, 1.633; p = 0.001) and NICU LOS 8.955 days less (95% CI: -14.399, -3.511; p = 0.001) than neonates exposed to methadone.

Conclusions

Neonates with in-utero exposure to buprenorphine/naloxone had significantly lower NOWS scores and shorter NICU LOS compared to neonates with in-utero exposure to methadone. These findings demonstrate that buprenorphine/naloxone is potentially a more favorable treatment for the reduction in metrics representing adverse neonatal outcomes in pregnant people with OUD than methadone.

## Introduction

Opioid abuse continues to be a major public health concern in the United States. The 2016 National Survey on Drug Use and Health found that 11.8 million Americans aged 12 or older reported misusing opioids [1]. Similarly, opioid exposure by pregnant people has remained a nationwide issue, with increasing prevalence mirroring that of the general population [2]. Between 2010 and 2017, there was a 131% increase in the recording of opioid exposure during pregnancy [3].

Opioid exposure during pregnancy affects fetal health, both in utero and perinatally. The complications of opioid exposure during pregnancy extend beyond newborn withdrawal, with prenatal opioid exposure associated with an increased risk of oligohydramnios, intrauterine growth restriction, premature birth, stillbirth, and neonatal opioid withdrawal syndrome (NOWS) [4-6]. NOWS is a complication of maternal opioid exposure that can present as a constellation of symptoms causing central nervous system irritability, gastrointestinal dysfunction, and autonomic activation [7]. Symptoms are assigned a number ranging from 1 to 4 depending on the severity of the symptom and a score is calculated to guide treatment.

The increasing rate of opioid exposure in pregnancy is associated with health system-level changes. Specifically, according to the Healthcare Cost and Utilization Project (HCUP), between 2010 and 2018, the prevalence of NOWS in newborns hospitalized in the United States increased from 4.0 to 6.8 per 1,000 hospital births. In addition, the average length of stay (LOS) for neonates with NOWS was approximately 10 days longer than that of other newborns. Furthermore, hospitalization with a diagnosis of NOWS was associated with an inflation-adjusted cost increase of approximately $7,100 to $7,900 [8].

Meanwhile, Michigan statistics roughly reflect national trends. Between 2010 and 2019, the rate of newborns admitted to the hospital with a diagnosis of NOWS increased from 3.6 to 6.0 per 1,000, with a high of 8.3 in 2015. LOS among newborns with NOWS was approximately seven to nine days longer, and there was a $6,200 to $8,900 increase in inflation-adjusted expenditure among these patients [8]. These statistics demonstrate the urgent need to address opioid exposure during pregnancy given the increasing prevalence of NOWS, the increased length of hospitalization of neonates affected by NOWS, and the significant increase in the cost of care for these patients that ultimately affects the entire US healthcare system, including Michigan’s infrastructure.

Medications for opioid use disorder (MOUD) is part of the treatment regimen used to treat opioid use disorder (OUD). In addition to counseling and behavioral therapies, MOUD involves the administration of long-acting opioid agonists or antagonists such as methadone, naltrexone, buprenorphine, or combination buprenorphine/naloxone. Meanwhile, the Centers for Disease Control and Prevention (CDC) recommends both methadone and buprenorphine as first-line treatment for OUD in pregnant people [9].

Studies that compared neonates with in-utero exposure to buprenorphine with neonates with in-utero exposure to methadone found that neonates exposed to buprenorphine had improved outcomes, specifically decreased incidence of NOWS [10-14], lower peak NOWS score [13], larger head circumference [10,15,16], higher birth weight [10,15,16], longer length [16], decreased likelihood of needing pharmacological treatment [12,13,17], reduced total amount of morphine used for treatment [13], decreased length of treatment [15], decreased need for additional therapies such as phenobarbital [15], reduced duration of NOWS [13], and shorter length of hospital stay [13,15,17]. Studies have hypothesized that buprenorphine/naloxone would have similar outcomes [18]. Early reports have shown that the incidence of NOWS is significantly higher in those exposed to buprenorphine compared to buprenorphine/naloxone [19]. Therefore, research on buprenorphine/naloxone is critical to help guide clinical practice.

In non-pregnant individuals, buprenorphine/naloxone has been preferred for MOUD due to its reduced abuse potential. However, it is not recommended to initiate the use of buprenorphine/naloxone in pregnancy due to the risk of inducing withdrawal because buprenorphine is a partial agonist [9,20,21]. Much of the current literature indicates potentially improved outcomes in neonates with in-utero exposure to buprenorphine/naloxone. However, there is not enough data to delineate a clear recommendation [18,19,22-26]. Yet, a recent Canadian study of 2,173 pregnant people, who received at least one opioid agonist treatment in pregnancy, found lower odds of preterm delivery, lower odds of disorders related to being small for gestational age (SGA) or low birth weight, and lower odds of NOWS in neonates with in-utero exposure to buprenorphine/naloxone or slow-release oral morphine (SROM) compared to neonates with in-utero exposure to methadone [27]. However, without a strict definition of study groups, a direct comparison of buprenorphine/naloxone and methadone is difficult.

Moreover, many of the previously cited studies evaluate a homogenous population, have limited sample sizes, recruit from a confined geographic location (particularly in urban areas), and are designed with a limited number of outcome variables. The rate of NOWS in rural communities in 2016 was 10.6 per 1,000 births compared to 6.2 per 1,000 births in urban communities [28]. The rate of NOWS in 2016 was also the highest in infants covered by Medicaid [28].

As such, the goal of the current study is to expand upon the current literature by evaluating a large, heterogenous population, in both rural and urban locations, against a wide array of NOWS parameters. The conclusions from this study have the potential to guide physicians and public health professionals in choosing the proper treatment modality for reducing adverse neonatal outcomes in people with OUD during their pregnancy.

## Materials and methods

Institutional Review Board (IRB) approval for this study was received from two medical centers’ IRBs and the Central Michigan University IRB committee (1420661-15).

Participants

A retrospective chart review evaluating female patients aged 18 years and older diagnosed with OUD and receiving methadone or buprenorphine/naloxone as MOUD during their pregnancy, resulting in exposure of the substances to their neonates, was conducted. Data from electronic medical records (EMR) were extracted from two academic medical centers from individuals who delivered between October 15, 2008, and October 15, 2019: one serving rural mid-Michigan, and the other serving urban southeast Michigan. Subjects were identified using International Classification of Diseases (ICD) 9 (648.33, 304.00) and ICD 10 (099.320, F11.20) codes for pregnancy, opioid addiction, and maintenance therapy. Pregnant people were identified at the time of delivery. MOUD treatment modality of buprenorphine/naloxone or methadone was obtained by the delivering provider. Where possible, prenatal medical records were reviewed to verify MOUD treatment modality. Furthermore, the charts of the pregnant individuals with OUD, as defined by the ICD 9 and 10 codes above, were associated with the charts of the child born during the period of our chart review to assess the outcomes of the neonates.

Data collection

A data collection sheet was produced in RedCap^©^ Software. A data dictionary was used to standardize data collection between trained study personnel and improve the final interpretation of data. Data collectors were trained in RedCap^©^ and the respective institutions’ EMRs. Three reviewers manually inputted the data into RedCap^©^ and completed randomized quality-controlled reviews of the data. If required, a fourth reviewer verified the data.

Metrics collected from the mothers’ medical charts included demographics, maternal health, prenatal care, and MOUD modality. Demographic data included zip code, health insurance status, race/ethnicity, and age. Maternal health indicators included self-reported tobacco smoking quantity (packs per day), multi-drug use, and alcohol use; comorbidities including diabetes, hepatitis, depression, asthma, obesity, bipolar disorder, and autoimmune diseases as indicated in their medical record; parity status; gestational age at delivery; delivery complications (defined by diagnosis-related group codes indicating complication); and LOS. Prenatal care, if undergone at all, was identified as the point of initiation of prenatal care during the pregnancy. Mental health conditions were included if there was a diagnosis in the medical record. Furthermore, we also collected the Area of Deprivation Index (ADI) state ranking based on the zip code of the home address associated with the mother’s medical chart.

Data collected from the neonates’ charts included sex, APGAR scores (at one and five minutes), head circumference, birth weight, length, NOWS status, peak NOWS score (as defined by the Finnegan Neonatal Abstinence Scoring System, utilized by both institutions), and length of newborn hospital stay [29]. Data from medical notes included free text and drop-down box information.

Study outcomes and data analysis

The main study outcomes of this investigation were to compare the incidence of NOWS, peak Finnegan score for NOWS, length of hospital stay of the neonate, APGAR scores at one and five minutes, and neonatal size parameters (head circumference, birth weight, and length). We included all neonates with in-utero exposure to buprenorphine/naloxone or methadone for our main study variables. For our main study variables, we included low NOWS scores in our analyses because we believed excluding them would introduce a data bias.

Demographic and delivery characteristics were compared between the buprenorphine/naloxone and methadone groups using chi-square analysis and t-tests. Furthermore, a series of multiple linear regression analyses were conducted to examine the effect of the type of therapy on each outcome after controlling for the mother’s socioeconomic, demographic, and delivery characteristics. More specifically, in our multiple regression, we controlled for maternal age, alcohol use during pregnancy, race, tobacco smoking quantity, ADI state ranking by zip code, medical insurance coverage, polysubstance use, parity status, prenatal care status, complications at delivery, gestational age, and maternal comorbidities of diabetes, hepatitis, depression, asthma, obesity, bipolar disorder, and autoimmune diseases, which were chosen based on prior research and theoretical risk on fetal outcomes [24,30,31]. We used appropriate measures of effect, p-value less than or equal to 0.05 and 95% confidence intervals (CIs), to determine significance.

## Results

Maternal characteristics

A total of 331 maternal medical records from two medical centers in Michigan, one rural and one urban/metropolitan, were evaluated. Of these records, 99 pregnant people were treated with buprenorphine/naloxone and 232 were treated with methadone. There was a significant difference (p < 0.05) in the mean maternal age between the buprenorphine/naloxone group and the methadone group, with values being 29.62 years (standard deviation (SD) = 4.5) and 28.16 years (SD = 4.8), respectively (Table 1). The only other significantly different maternal factor was hepatitis (buprenorphine/naloxone = 24.2%; methadone = 36.2%; p < 0.05) (Table 1). Other baseline maternal characteristics of interest were similar in each group.

**Table 1:** Maternal sample characteristics and comparisons of buprenorphine/naloxone vs. methadone group. *p-value <0.05; **p-value <0.01; ***p-value <0.001. All percentages are valid percentages. SD: standard deviation; BMI: body mass index

	Buprenorphine/Naloxone	Methadone	P-value
	(N = 99)	(N = 232)	
Maternal characteristics			
Age			
Mean (SD)	29.62 (4.5)	28.16 (4.8)	<0.05*
Minimum, Maximum	21–41	20–42	
Substance use			
Smoking status (N)	82.8% (N = 82)	83.5% (193)	0.87
Alcohol use (N)	6.1% (6)	10.4% (24)	0.21
Marijuana use (N)	37.7% (26)	40.3% (58)	0.71
Poly-substance use (N)	51.5% (51)	62.8% (145)	0.06
Race			
Hispanic (N)	2.1% (2)	2.6% (6)	0.60
Black/African American (N)	4.1% (4)	3.1% (7)	0.83
White (N)	92.8% (90)	91.7% (210)	0.74
Maternal comorbidities			
Diabetes (N)	4.0% (4)	2.6% (6)	0.48
Hepatitis (N)	24.2% (24)	36.2% (84)	<0.05*
Depression (N)	38.4% (38)	36.6% (85)	0.76
Asthma (N)	7.1% (7)	12.5% (29)	0.15
Bipolar disorder (N)	18.2% (18)	12.1% (28)	0.14
Schizophrenia (N)	0.0% (0)	0.0% (0)	-
Obesity (BMI > 30) (N)	2.0% (2)	1.7% (4)	0.85
Metabolic syndrome (N)	0.0% (0)	0.0% (0)	-
Autoimmune disease (N)	2.0% (2)	2.2% (5)	0.93
Other psychiatric disorder (N)	33.3% (33)	25.4% (59)	0.14
Insurance status			
Medicaid (N)	79.8% (79)	81.0% (188)	0.79
Private insurance (N)	21.2% (21)	18.5% (43)	0.57
Medical complications at delivery (N)	27.6% (27)	25.7% (59)	0.72
Gestational age in weeks (SD)	38.20 (1.78)	37.05 (3.80)	0.005**
Prenatal care (N)	78.1 (75)	74.9 (170)	0.53

Neonatal characteristics

The mean LOS of neonates with in-utero exposure to buprenorphine/naloxone versus methadone was significantly different, with 11.78 days (SD = 9.93) compared to 21.34 days (SD = 22.08), respectively (p < 0.001) (Table 2). NOWS incidence was 70.1% for the buprenorphine/naloxone group compared to 78.8% for the methadone group (p = 0.06). Furthermore, there were significant differences at baseline for NOWS peak score (buprenorphine/naloxone = 9.49 (SD = 4.26); methadone = 11.61 (SD = 3.84); p < 0.001), birth head circumference (cm) (buprenorphine/naloxone = 33.09 (SD = 3.44); methadone = 32.29 (SD = 3.84); p < 0.05), birth weight (g) (buprenorphine/naloxone = 2,988.6 (SD = 564.75); methadone = 2,750.88 (SD = 710.00); p < 0.05), and APGAR score at one minute (buprenorphine/naloxone = 8.21 (SD = 1.36); methadone = 7.83 (SD = 1.65); p < 0.05) (Table 2). Other baseline neonatal characteristics were similar between the two groups.

**Table 2:** Sample characteristics and comparisons of buprenorphine/naloxone vs. methadone group for neonatal outcomes. *p-value <0.05; ***p-value <0.01. All percentages are valid percentages. SD: standard deviation; IQR: interquartile range: NOWS: neonatal opioid withdrawal syndrome

	Buprenorphine/Naloxone	Methadone	P-value
	(N = 99)	(N = 232)	
Neonatal characteristics			
Birth head circumference (cm)			
Mean (SD)	33.09 (3.44)	32.29 (3.84)	<0.05*
Minimum, Maximum	13.38–38.50	12.40–37.0	
Birth weight (g)			
Mean (SD)	2,988.6 (564.75)	2,750.88 (710.85)	<0.01*
IQR	2,602–3,365	2,437–3,220	
Birth length (cm)			
Mean (SD)	47.83 (4.41)	49.26 (28.81)	0.24
IQR	46.00–50.00	45.7–49.7	
APGAR at 1 minute			
Mean (SD)	8.21 (1.36)	7.83 (1.84)	<0.05*
Minimum, Maximum	1.0–9.5	0–9.0	
APGAR at 5 minutes			
Mean (SD)	8.84 (0.65)	8.52 (1.65)	<0.05*
Minimum, Maximum	5–10	0–10	
NOWS peak score			
Mean (SD)	9.49 (4.26)	11.61 (3.84)	<0.001***
Min, Max	0–18	3–27	
NOWS incidence (%)	68 (70.1%)	175 (78.8%)	=0.06
Length of stay			
Mean (SD)	11.78 (9.93)	21.35 (22.08)	<0.001***
IQR	4–16.9	6–29.4	

Multivariate analysis

Our multivariate linear regression found NOWS peak score and (LOS) to be significantly different for neonates with in-utero exposure to buprenorphine/naloxone versus neonates with in-utero exposure to methadone (Table 3). Specifically, our adjusted analysis found that neonates with in-utero exposure to buprenorphine/naloxone had a NOWS peak score of 3.079 points less than those with in-utero exposure to methadone (95% CI = -4.525, -1.633; p = 0.001). LOS of neonates exposed to buprenorphine/naloxone was 8.955 days less than neonates exposed to methadone (95% CI = -14.399, -3.511; p = 0.001) (Table 3). There were no significant differences found in our adjusted analysis between neonatal outcomes of APGAR scores at one and five minutes, head circumference, or length for neonates with in-utero exposure to buprenorphine/naloxone or methadone (p > 0.05) (Table 3).

**Table 3:** Regression analyses for neonatal outcomes. *p-value <0.05; **p-value <0.01. Each regression was controlled for maternal alcohol use, marijuana use, maternal age at delivery, race, smoking quantity, ADI state ranking, ADI national rank mean of the Zip, insurance, poly-drug use, gravida, para, gestational age in weeks, medical complications at delivery, prenatal care, diabetes, hepatitis, depression, asthma, bipolar disorder, obesity, autoimmune disorder, and psychiatric disorder. The full regression table with all variables can be viewed in the Appendix. CI: confidence interval; NOWS: neonatal opioid withdrawal syndrome; ADI: Area of Deprivation Index

Outcome	Coefficient	95% CI	P-value
NOWS peak score	-3.079	(-4.525,-1.633)	0.001**
Head circumference (cm)	-0.636	(-1.954, 0.683)	0.342
Weight (g)	67.748	(-68.338, 203.835)	0.327
Length (cm)	0.672	(-0.756, 2.100)	0.353
Length of stay	-8.955	(-14.399, -3.511)	0.001**
APGAR at 1 minute	-0.034	(-0.599, 0.532)	0.906
APGAR at 5 minutes	0.017	(-0.371, 0.404)	0.932

## Discussion

The present study examined EMR data from two Michigan health systems, one serving rural mid- and upper-Michigan and the other serving southeast urban Michigan. We found that neonates with in-utero exposure to buprenorphine/naloxone had significantly lower NOWS scores and shorter LOS compared to neonates with in-utero exposure to methadone. However, there were no significant differences in outcomes for APGAR scores at one and five minutes, head circumference, or neonatal length.

The results of the present study demonstrate that buprenorphine/naloxone has the potential to be a more effective treatment in reducing adverse neonatal outcomes. This study encompasses a large and heterogenous population of individuals who had OUD during pregnancy and were on MOUD. By including a large sample of rural and urban individuals and a greater proportion of minority populations compared to previous studies, this study is more translatable and provides valuable information to guide medical decision-making in diverse populations.

Further, buprenorphine/naloxone is an attractive MOUD choice because it can be dispensed in an office-based setting due to its lower risk of overdose and improved safety profile [32]. Meanwhile, methadone must be dispensed daily from a Substance Abuse and Mental Health Services Administration (SAMHSA)-certified clinic. This makes buprenorphine/naloxone a valuable choice, especially for patients who have difficulty accessing methadone clinics in lieu of a physician’s office. New, effective, and safe MOUD modalities that also increase access to treatment will ultimately improve compliance and health outcomes in both pregnant people and their children. However, because methadone is an approved medication for OUD in pregnancy, it would require the prescribing provider to provide off-label, not-approved-for, treatment of buprenorphine/naloxone for pregnant mothers using a risk-benefit ratio and shared decision-making strategies.

This study builds upon earlier studies that similarly demonstrated significantly lower NOWS scores among neonates with in-utero exposure to buprenorphine/naloxone compared to neonates with in-utero exposure to methadone. However, those studies did so with much smaller sample sizes in comparison to the present study [23,24,26]. This study also found significant decreases in LOS in neonates with in-utero exposure to buprenorphine/naloxone compared to neonates with in-utero exposure to methadone. Data from earlier studies reported varying conclusions on LOS [24,25]. For instance, Wiegand et al. reported that neonates exposed to buprenorphine/naloxone had significantly shorter LOS compared to neonates exposed to methadone (5.6 ± 5 compared with 9.8 ± 7.4 days; p = 0.02). Another study published in 2014 performed by Gawronski et al. reported a similar LOS in neonates exposed to buprenorphine/naloxone and neonates exposed to methadone (9 ± 6 compared with 10 ± 8 days; p > 0.05) [24,25]. Our findings are also consistent with investigations analyzing buprenorphine alone in comparison to methadone [15,17]. These data demonstrate the importance of further studies that examine outcomes of neonates exposed to different MOUD modalities available, especially in buprenorphine/naloxone-exposed populations as the present study evaluated.

The present study is not without limitations. The main limitations of this study were the retrospective study design and the subjectivity of the data in the EMR. Using a retrospective chart review means data collection is limited to the information present in the individual health records, which does not allow us to qualify the data nor control how the data was input. The subjectivity of retrospective chart reviews also means we are analyzing self-reported participant data without the ability to contextualize the information or contact participants for clarification. Furthermore, we must consider the social pressures that exist in every stage of pregnancy, and especially at the time of delivery, which may result in minimization of other substance use, for example, for fear of negative interactions with child protective services. Additionally, data can be perceived differently between data extractors, which may affect both the accuracy and precision of the data. To compete against this, we implemented a standardized collection process with a data dictionary and the REDcap platform, as well as randomized quality control reviews during data abstraction, were used to limit further variability in the data. Despite this, outliers were present in the neonatal birth weight variable, which could be attributed to data extraction errors. On re-analysis, removing these values resulted in no significant values in the median birth weight. The presence of these values requires a cautious interpretation of the conclusions associated with these results.

Furthermore, we did not control several variables such as severity, dose/duration of MOUD treatment, specific socioeconomic factors, other mental health medications, or contextual maternal variables (e.g., domestic violence and difficulty obtaining care) which could be confounding variables and could be useful in future investigations to contextualize the outcomes. Further studies should define a study design that considers these variables. Finally, how the drugs are dispensed to patients (i.e., methadone must be in a clinic setting, while buprenorphine/naloxone is outpatient) introduces both a selection bias and increased monitoring effect, where patients in the methadone group are selected for their treatment group because they have underlying medical, obstetric, or psychiatric concerns that require more routine monitoring.

## Conclusions

Despite the limitations, our analysis determined that neonates with in-utero exposure to buprenorphine/naloxone had significantly lower NOWS scores and shorter LOS compared to neonates with in-utero exposure to methadone. This investigation is the largest known investigation in Michigan to date comparing outcomes in neonates with in-utero exposure to buprenorphine/naloxone and methadone among both rural and urban populations. This adds to the literature and improves evidence-based medicine practices. The results of the present study demonstrate that buprenorphine/naloxone has the potential to be a more effective treatment in reducing adverse neonatal outcomes in mothers who have OUD during pregnancy. Future investigations of this topic should incorporate a randomized prospective design to ascertain the causality of the exposures more adequately.

## Appendices

**Table 4:** Regression analyses for neonatal outcomes, full variable list. *p-value <0.05; **p-value <0.01; ***p-value <0.001. ADI: Area of Deprivation Index

	NOWS peak score		APGAR at 1 minute		APGAR at 5 minutes		Length of stay		Head circumference (cm)		Weight (g)		Length (cm)	
Variables	Coefficient	P-value	Coefficient	P-value	Coefficient	P-value	Coefficient	P-value	Coefficient	P-value	Coefficient	P-value	Coefficient	P-value
Maternal drug buprenorphine/naloxone	-3.079	<0.001***	-0.034	0.906	0.017	0.932	-8.955	0.001***	-0.636	0.342	67.748	0.327	0.672	0.353
Maternal alcohol use	1.796	0.070	0.012	0.975	0.099	0.711	7.592	0.044*	0.054	0.954	-4.222	0.964	-0.567	0.529
Marijuana use	-0.290	0.690	0.178	0.532	-0.045	0.816	-3.691	0.180	-0.156	0.810	-74.379	0.279	-0.467	0.488
Maternal age at delivery	-0.012	0.873	0.036	0.227	0.035	0.082	-0.285	0.311	0.002	0.982	-16.050	0.025*	0.073	0.289
Race is non-white	-3.004	0.017*	-0.294	0.510	-0.490	0.110	-6.151	0.155	1.331	0.226	39.433	0.713	-1.277	0.199
Smoking quantity	2.341	0.042*	-0.940	0.031*	-0.342	0.250	7.014	0.097	-0.757	0.448	-104.033	0.318	-0.574	0.576
ADI state ranking	3.068	0.251	0.117	0.911	-0.002	0.998	8.457	0.406	-0.479	0.845	533.568	0.036*	-1.277	0.601
ADI national rank mean of the zip	-0.398	0.251	-0.008	0.956	0.009	0.925	-1.054	0.428	0.038	0.906	-66.841	0.044*	0.217	0.497
Insurance (Medicaid = ref)	2.039	0.046*	-0.252	0.520	-0.447	0.098	-2.204	0.560	0.322	0.715	-49.771	0.598	-0.063	0.944
Poly-drug use	-0.702	0.351	0.022	0.941	-0.009	0.966	2.396	0.395	-0.294	0.658	-8.297	0.907	1.362	0.047*
Gravida	0.359	0.258	-0.089	0.475	-0.163	0.056	-2.710	0.032*	-0.421	0.159	30.240	0.312	0.366	0.249
Para	-0.295	0.458	0.140	0.364	0.246	0.021*	2.971	0.056	0.224	0.547	-31.998	0.389	-0.802	0.044*
Gestational age (in weeks)	0.435	0.011*	0.294	<0.000***	0.293	<0.000**	-0.345	0.449	0.492	<0.000***	152.893	<0.000***	0.850	<0.000***
Medical complications at delivery	1.671	0.062	-0.861	0.014*	-0.338	0.156	3.382	0.319	-0.055	0.946	-92.531	0.269	-1.489	0.065
Prenatal care (Ref = yes)	-0.466	0.512	-0.435	0.120	-0.328	0.085	1.247	0.345	-0.907	0.167	-25.911	0.698	0.564	0.403
Diabetes	2.391	0.168	0.836	0.251	0.248	0.619	2.640	0.707	2.229	0.172	60.812	0.728	-6.575	<0.001***
Hepatitis	-0.637	0.339	0.017	0.949	-0.086	0.637	-0.877	0.732	0.585	0.365	-75.314	0.239	-0.030	0.963
Depression	0.004	0.996	-0.173	0.540	0.014	0.941	-2.576	0.342	0.705	0.285	66.971	0.318	0.013	0.985
Asthma	0.465	0.662	-0.600	0.183	0.002	0.995	-4.117	0.343	-1.854	0.073	-80.537	0.457	1.347	0.231
Bipolar disorder	0.784	0.376	0.310	0.396	0.186	0.455	-0.153	0.966	0.660	0.430	-174.745	0.048*	0.296	0.743
Obesity	1.794	0.339	-0.364	0.617	-0.096	0.847	-4.167	0.552	-2.430	0.136	328.606	0.062	4.764	0.011**
Autoimmune disease	3.287	0.129	-0.099	0.901	0.690	0.207	16.864	0.029*	0.078	0.965	-218.773	0.255	-0.857	0.630
Psychiatric disorder	-0.477	0.546	-0.136	0.657	-0.083	0.695	3.265	0.277	0.240	0.739	144.172	0.053	0.236	0.756
